# Effects of aquatic therapy vs. standard care on gluteal muscle morphology and function in individuals with chronic low back pain: a randomized controlled trial

**DOI:** 10.1186/s13102-026-01666-0

**Published:** 2026-04-06

**Authors:** Chanelle Montpetit, Nicolas Vaillancourt, Brent Rosenstein, Evert Onno Wesselink, Geoffrey Dover, Christina Weiss, Lee Ann Papula, Antonys Melek, Kenneth Arnold Weber, James M. Elliott, Maryse Fortin

**Affiliations:** 1 Department of Health, Kinesiology and Applied Physiology, Concordia University, Montreal, QC Canada; 2 CRIR – Centre de réadaptation Constance-Lethbridge du CIUSSS COMTL, Montreal, QC Canada; 3 School of Health, Concordia University, Montreal, QC Canada; 4 School of Medicine, Division of Pain Medicine, Department of Anesthesiology, Perioperative and Pain Medicine, Stanford University, Palo Alto, CA USA; 5 Faculty of Medicine and Health, School of Health Sciences, The Kolling Institute, University of Sydney, Sydney, NSW Australia; 6 Northern Sydney Local Health District, St. Leonards, NSW Australia

**Keywords:** Chronic low back pain, Aquatic therapy, Gluteal muscles, Magnetic resonance imaging, Morphology, Function

## Abstract

**Background:**

Muscular dysfunction of the lumbar-pelvic-hip complex is a hallmark of chronic low back pain (cLBP). This dysfunction is partly driven by muscle morphometry and composition, highlighting their importance in cLBP. Exercise-based interventions are commonly used in cLBP management; however, the extent to which different therapeutic approaches influence gluteal muscle health remains unclear. Aquatic therapy may offer a distinct therapeutic environment by reducing spinal loading while facilitating progressive muscle activation.

**Objectives:**

This study aimed to (1) investigate the effects of aquatic therapy (AT) versus standard care (SC) on gluteal muscle size, intramuscular fat (IMF) and strength in patients with cLBP and (2) investigate whether positive changes in gluteal muscle health (i.e., size, composition, strength) are associated with concomitant improvements in patient-oriented outcomes.

**Methods:**

This randomized controlled trial included 34 participants with moderate-to-severe cLBP. The participants were randomized to either AT (*n* = 18) or SC (*n* = 16). Both groups completed a 10-week supervised intervention program consisting of 2 sessions per week. The baseline and postintervention assessments included pelvic magnetic resonance imaging, gluteal strength testing, and patient-reported questionnaires. Changes in muscle-related and patient-oriented outcomes within and between groups were analyzed using repeated-measures analysis of covariance, with baseline disability as a covariate.

**Results:**

Repeated measures analysis of covariance revealed no significant changes in gluteal muscle volume in either group. Significant decreases in IMF were observed in the gluteus maximus (-2.56 [95%CI: -4.47 to -0.64], *p* = 0.01) and gluteus medius (-1.00 [95%CI: -1.97 to -0.04], *p* = 0.04) in the AT group, with no significant changes in the SC group. Both groups demonstrated significant increases in gluteal maximus (AT:55.20 [95%CI: 31.84 to 78.55], *p* < 0.001; SC:41.45 [95%CI: 16.52 to 66.38], *p* = 0.002) and medius (AT:72.34 [95%CI: 44.62 to 100.05], *p* < 0.001; SC:43.91 [95%CI: 14.32 to 73.49], *p* = 0.005) strength. Improvements in gluteus IMF were moderately correlated with increases in physical quality of life (gluteus maximus: *r*=-0.33; gluteus medius: *r*=-0.31) and decreases in pain catastrophizing (gluteus minimus: *r* = 0.39).

**Conclusion:**

The AT group demonstrated significant improvements in strength and IMF content, suggesting potential musculoskeletal benefits health in individuals with cLBP.

**Trial registration:**

This study was registered at ClinicalTrials.gov (NCT05823857 on 10/04/2023).

**Supplementary Information:**

The online version contains supplementary material available at 10.1186/s13102-026-01666-0.

## Introduction

Low back pain is the most prevalent and persistent musculoskeletal disorder worldwide [1]. Up to 90% of the population will experience some form of low back pain during their lifetime [2, 3]. Since 1990, the global burden of disability due to low back pain has been rapidly increasing, resulting in billions of dollars in healthcare and societal costs in Canada each year [2–4]. Low back pain is typically classified as acute, subacute, or chronic [5]. While approximately 90% of cases resolve within 12 weeks without lasting impairments, the remaining 10% persist as chronic low back pain, which contributes disproportionately to the overall economic and societal burden [6, 7].

Muscular dysfunction of the lumbar-pelvic-hip complex is a hallmark of chronic low back pain [8]. Morphological alterations in the gluteal muscles, such as changes in the muscle cross-sectional area, volume or intramuscular fat, have been reported in individuals with chronic low back pain [9]. In addition to potential morphological changes, individuals with chronic low back pain are more likely to exhibit functional impairments in the gluteal muscles, including reduced strength and muscle activation [10–12]. Gluteal muscle dysfunction may lead to a lack of support of the trunk and pelvis, which is thought to play a role in the development or exacerbation of low back pain [13–16]. A key component of more effective assessment techniques and management of low back pain is determining the differences in gluteal morphology, composition, and function between those with chronic low back pain and those without [17].

The management of chronic low back pain is multifaceted, with exercise therapy playing a central role [5, 18, 19]. Exercise-based interventions have been shown to improve quality of life and reduce pain in individuals with chronic low back pain [20]. Given the growing evidence linking chronic low back pain and dysfunction of the hip, trunk, and paraspinal muscles, exercise interventions often target activation and strengthening of these muscle groups [21]. Aquatic therapy has been used for many years in the management of musculoskeletal conditions, including chronic low back pain, with reported positive effects on patient outcomes, such as improved function, increased spinal flexibility, and reduced work absenteeism [2]. The therapeutic effects of aquatic therapy are attributed to the physical properties of water, including buoyancy, hydrostatic pressure, viscosity, and thermodynamics [22]. Buoyancy reduces the stress on joints and muscles, hydrostatic pressure enables a greater range of motion by supporting the weight of the body, depth variations allow for the progression of resistance, and warm water increases muscle relaxation and efficiency [5, 8]. By utilizing the unique properties of water, a graded intervention can be created to address an individual patient’s needs and deficits. While exercise therapy is the most widely used form of conservative treatment for chronic low back pain, it remains unclear which form of exercise is the most effective for managing and treating this condition. Aquatic therapy has been reported to yield improvements equal to or greater than those of land-based exercise programs and may be particularly suitable for people with chronic low back pain who are in the early stages of rehabilitation, are fearful of movement or have difficulty performing land exercises [2]. However, whether aquatic therapy can lead to significant physiological adaptations in gluteal morphology, composition, and function has yet to be investigated.

The objectives of this study were to (1) investigate the effects of aquatic therapy versus standard care on gluteal muscle size, composition (i.e., intramuscular fat), and strength in individuals with chronic low back pain and (2) investigate whether positive changes in gluteal muscle health are associated with parallel improvements in patient-reported outcomes (e.g., pain and disability). We hypothesized that the aquatic therapy group would show significant improvements in gluteal muscle size, composition and strength compared with the standard care group and that these changes would be positively correlated with improvements in patient-reported outcomes.

## Methods

### Study design and setting

**Fig. 1:**
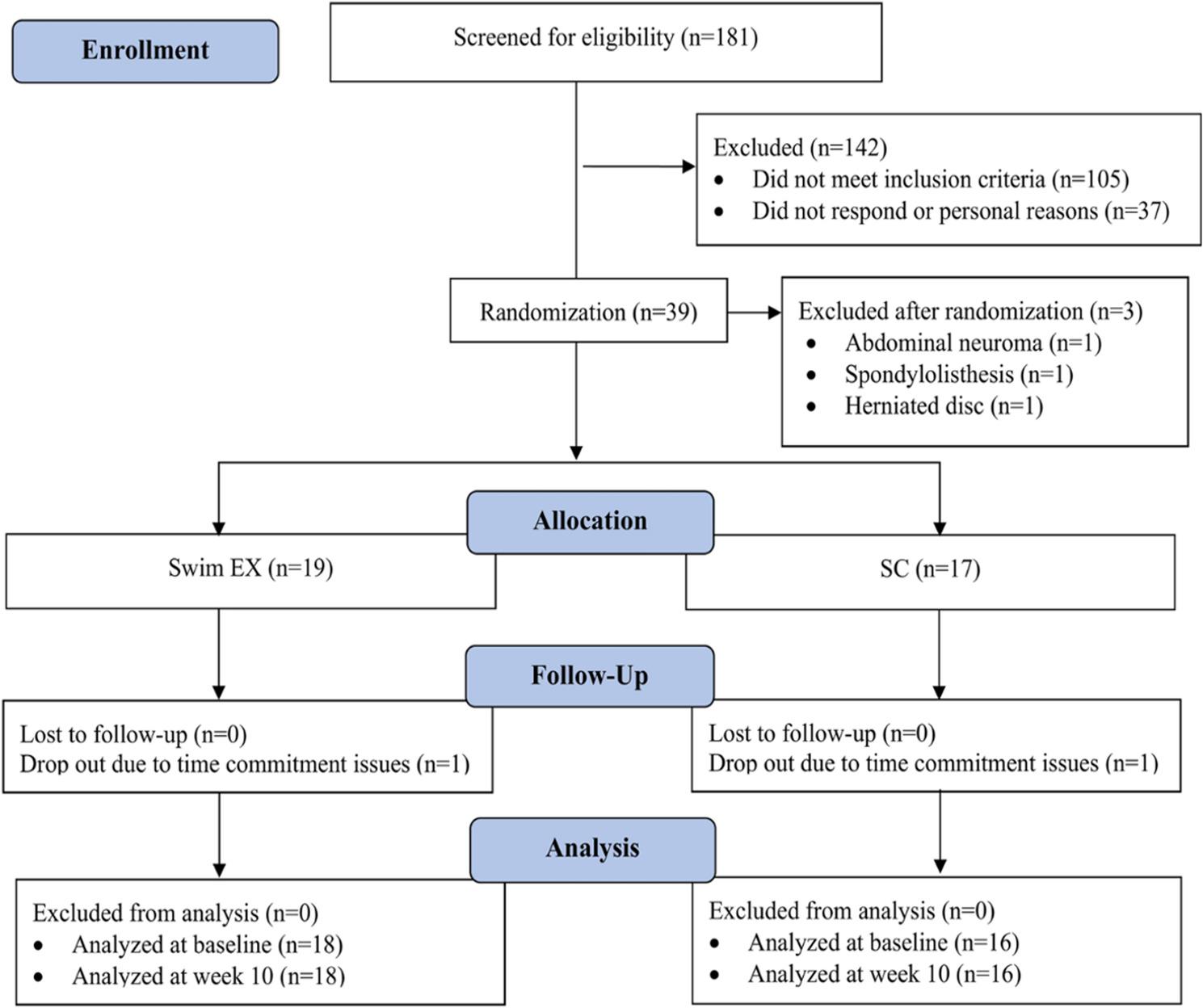
Consort flow diagram

This study was a secondary objective of a two-arm randomized controlled trial (RCT) that compared the effects of aquatic therapy and standard care on paraspinal muscle and psychological health in individuals with chronic low back pain [23, 24]. The RCT protocol was previously published [25], and the trial was prospectively registered (NCT05823857 on 10/04/2023). The study was conducted at the School of Health, Concordia University, and received approval from the Central Ethics Research Committee of the Quebec Minister of Health and Social Services (#CCER-21-22-35). Written informed consent was obtained from each participant. The study was reported in accordance with the CONSORT statement [26]. The flowchart diagram is presented in Fig. 1.

### Participants

Individuals were eligible to participate in the trials if they met the following inclusion criteria: (1) nonspecific chronic low back pain lasting ≥ 3 months, (2) aged 18–65 years, (3) proficient in English or French, (4) actively seeking care for low back pain, (5) a score indicating moderate or severe disability on the modified Oswestry Disability Index [27], and (6) no engagement in core/gluteal muscle-specific training within 3 months prior to the trial. Exclusion criteria included: (1) signs of nerve root compression or reflex motor sign deficits (e.g., weakness, altered reflexes, or sensory changes), (2) previous spinal surgery or vertebral fractures, (3) major lumbar spine structural abnormalities (e.g., lumbar scoliosis > 10°, spondylolysis, spondylolisthesis), and (4) any health conditions precluding safe participation in exercise programs as determined by the Physical Activity Readiness Questionnaire [28]. Participants were withdrawn from the intervention if they missed more than 7 sessions (35% of the intervention). The participants were asked to refrain from receiving other types of treatment (e.g., exercise therapy, manual therapy, and massage therapy) and medication during the intervention period, although this did not hinder participation. The participants were also asked to report any cointerventions or pain medication.

### Participant recruitment

Participants were recruited from the School of Health Athletic Therapy Clinic and through local university media advertising (e.g., email blast), as this approach has been shown to effectively recruit participants [21]. Participants who expressed interest in the study were contacted by a research team member to confirm eligibility and enrollment. Participant recruitment started in October 2022, and data collection was completed by December 2023.

### Randomization and blinding

The participants were randomly allocated (1:1) to one of two treatment groups via sealed, opaque, consecutively numbered envelopes. The envelopes were prepared by an independent individual not involved in the study, based on a computer-generated randomization sequence with permuted blocks. Eligible participants were randomized into either the aquatic therapy group or the standard care group. Blinding of therapists and participants was not feasible given the nature of the exercise interventions [29].

### Procedure

During the first session, the participants came to the School of Health to complete a general study consent form, a magnetic resonance imaging (MRI) consent form, and patient-reported questionnaires [30]. The participants also underwent baseline lumbopelvic MRI and gluteal muscle strength testing. The participants in both groups completed a 10-week intervention, consisting of two supervised 60-minute sessions per week. The training frequency aligns with previous exercise intervention research for individuals with chronic low back pain [31]. The 10-week duration was selected on the basis of evidence that training-induced muscle changes typically occur within this timeframe [32]. According to the American College of Sports Medicine, 24–36 h of rest is recommended between light or aerobic exercise sessions, whereas a minimum of 48 h is advised between resistance training sessions targeting the same muscle group [33]. Accordingly, sessions were scheduled 1–2 days apart during the week. All intervention sessions for both the aquatic therapy and standard care groups were conducted by a certified athletic therapist.

### Interventions

Each exercise intervention has been explained in detail in a previous publication [25]. Thus, only a summary of the interventions will be reported in the current study.

#### Aquatic therapy group

The aquatic therapy exercise program [25] was adapted from a recent intervention in adults with chronic low back pain [2] (see Additional file, Table 1). The aquatic therapy intervention took place in the School of Health Athletic Therapy SwimEx 700T pool (5.36 m length x 1.98 m width x 1.83 m depth). Participants in this group performed trunk and pelvis stabilization exercises based on a variety of aquatic exercises in different positions for coordinated contraction of the multifidus, transverse abdominus and gluteal muscles. The purpose of these exercises was to improve the strength and dynamic stability of the spine and pelvis in a functional but non-weight-bearing way. Each exercise was performed 10 times in a controlled manner. The exercises were progressed by adding a load to the muscles through resistance material (kickboard, dumbbell floats), external leverage at the limbs (hand paddles, ankle weights), and a water current. Each aquatic therapy session included a 10-minute warm-up, a 40-minute aquatic therapy session, and a 10-minute cool-down for a total of 60 min.

#### Standard care group

Participants in this group received standard treatment for low back pain. The standard care intervention took place at the School of Health Athletic Therapy Clinic and conditioning floor. During the first session, the athletic therapist completed a thorough assessment of the participant. The assessment included relevant background information (e.g., medical history, lifestyle assessment), postural assessment, gait and functional movement assessment, and physical examination. The Athletic Therapist administered a range of techniques, including stretching, manual spinal mobilizations, strengthening and stabilization exercises, aerobic conditioning, and modality application (e.g., ice, heat), based on their assessment findings. Each session lasted approximately 60 min. The standard care treatments were not regulated or standardized to mirror what happens in a rehabilitation clinic during low back pain treatment.

### Outcome measures

All outcome measures were collected at baseline and following the 10-week intervention for participants in both groups. The primary outcome measures included gluteal muscle size, composition (i.e., intramuscular fat), and strength. The secondary outcomes include changes in patient-reported outcomes (i.e., disability, pain, pain catastrophizing, kinesiophobia, anxiety, depression, insomnia).

####  Gluteal MRI

 All participants underwent a baseline lumbopelvic MRI evaluation before the beginning of the intervention via the PERFORM Centre’s 3-Tesla GE scanner (standard phased-array body coil with 16 channels, 4-mm slice thickness) to ensure that none of the exclusion criteria were met. Imaging sequences included the following: (1) Gluteal axial Lava-Flex (3D [three-dimensional] T_1_-weighted technique using Dixon fat-water separation to create multiple images from a single acquisition; field of view = 42 cm, slice thickness = 4 mm, matrix = 320 × 192, flip angle = 5°, TE = minimum full); and (2) Gluteal axial T_2_-weighted FRFSE (FOV = 42 cm, slice thickness = 4 mm, matrix = 384 × 256). These sequences were used to assess gluteal muscle morphology (i.e., size and composition). Bilateral automated segmentation of the regions of interest was performed via a convolutional neural network from the MuscleMap Toolbox [34, 35] along every slice from the most cranial aspect of the gluteal muscles to the most caudal slice, and the 3D volume of the right and left was calculated for each muscle, which is more accurate than assessing size from a single axial slice [34, 35]. One assessor (CM) then manually corrected the segmentations as necessary. The measurements obtained included the volume (cm^3^) and percentage of intramuscular fat (%IMF). Additionally, the spatial distribution of the %IMF was assessed across each gluteal muscle, allowing visualization of whether intramuscular fat was concentrated in specific regions or more evenly dispersed. Here, we automatically divided the muscles into two tiles on both sides to evaluate the spatial distribution of intramuscular fat across the anterior and posterior regions, as well as from medial to lateral (Refer to Fig. 3). We used a standard orthogonal plane, where anterior/posterior and medial/lateral refer to the anatomical orientation. This method has been shown to be reliable [36].

#### Gluteal muscle strength

A hand-held dynamometer (micro FET2; Hoggan Health Industries) was used to assess gluteal muscle strength. The participants were placed on a therapy table in a prone position to assess gluteus maximus muscle strength, with the knee at 90 degrees of flexion and the thigh/hip slightly extended (e.g., off the table). The participants were instructed to maintain this position for 3 s, creating an isometric contraction in the form of a “make” test, and were asked to exert a maximal force against the hand-held dynamometer. The measurements were recorded in newton/torque. All participants had a practice trial, and then 3 measurements were obtained on each side, with a 1-minute break between measurements. The means were used in the analysis. Similarly, patients were placed in a side-lying position with the leg abducted and slightly extended to test the gluteus medius muscle. The participants were again instructed to maintain this position for 3 s while exerting a maximal force against the hand-held dynamometer. Three measurements will be acquired on each side. Two certified athletic therapists acquired the measurements. Handheld dynamometry is a valid [37] and highly reliable tool for assessing gluteus muscle strength [38] and has been recommended as a practical standard for clinical settings.

#### Patient-reported questionnaires

A series of patient-reported outcome measures (i.e., questionnaires), were used to assess disability, pain intensity, health-related quality of life, pain-related fear (kinesiophobia, catastrophizing), anxiety, depression, and sleep quality. Disability was assessed via the modified Oswestry Low Back Pain Disability Index (ODI). Pain intensity was measured via the Numerical Pain Rating Scale (NPRS), in which participants rated their average low back pain over the past seven days on a scale from 0 (no pain) to 10 (worst imaginable pain). Health-related quality of life was evaluated via the Short Form-12 Health Survey (SF-12), which includes both a physical component and mental component summary. Pain-related fears were assessed via the Tampa Scale of Kinesiophobia (TSK) and the Pain Catastrophizing Scale (PCS). Anxiety and depression were assessed via the Hospital Anxiety and Depression Scale (HADS). Sleep quality was evaluated via the Insomnia Severity Index (ISI). All patient-reported questionnaires have demonstrated strong test-retest reliability and have been validated for use in individuals with chronic low back pain [39–44].

### Sample size justification

As this was a secondary analysis [23], the sample size calculation was based on prior studies examining the effects of exercise interventions on multifidus muscle size [45–47]. Using a mean effect size of 0.73, the required sample size was determined at a significance level of α = 0.05 and 80% power. Based on these calculations, a total of 34 participants were recruited for the study (17 in each group).

### Statistical analysis

Demographic characteristics were compared between groups via independent t-tests for continuous variables and chi-square tests for categorical variables. Primary and secondary outcomes were initially evaluated with descriptive statistics. Mixed repeated-measures analyses of covariance (ANCOVA) were conducted to assess changes in gluteal muscle morphology and strength over time, with treatment group (aquatic therapy vs. standard care) included as the between-subjects factor, time (baseline and post-intervention) as the within-subjects factor, and baseline disability (ODI) as a covariate; as well as the group × time interaction were examined. Assumptions of normality, homogeneity of variance, and sphericity were confirmed using the Kolmogorov-Smirnov and Shapiro-Wilk tests, Levene’s test, and Mauchly’s test of sphericity, respectively. Pearson correlation analyses were conducted to explore associations between changes in gluteal muscle morphology and changes in patient-reported outcomes, including disability, pain, quality of life, pain-related fear, anxiety, depression, and sleep quality. Muscle morphology outcomes were aggregated across all the magnetic resonance images of the gluteal region. For each level, the left and right 3D muscle volumes, percent intramuscular fat (%IMF) and spatial distribution were averaged and summed to provide composite measures of gluteal muscle volume and composition. Correlation strengths were interpreted according to Cohen’s criteria: small/weak (*r* = 0.10–0.29), moderate (*r* = 0.30–0.49), and strong (*r* ≥ 0.50) [48]. All analyses were conducted using an intention-to-treat approach. Statistical analyses were performed using IBM SPSS Statistics version 29.0 (IBM Corp., Armonk, NY, USA); a p-value of < 0.05 was considered statistically significant.

## Results

### Participants

Participant baseline characteristics are presented in Table 1. A total of 39 participants with chronic low back pain consented to participate at baseline, and 34 completed the 10-week follow-up. In the standard care group, three participants were excluded after randomization due to baseline MRI findings, and one withdrew due to time constraints. In the aquatic therapy group, one participant also withdrew due to time constraints. Both the aquatic therapy and standard care groups demonstrated high adherence to the intervention, with an average attendance of 17.56 out of 20 sessions (87.8%). No participants reported using opioid medications during the intervention period. Reported co-interventions included exercise (17.6%), massage therapy (5.9%), nonsteroidal anti-inflammatory drugs (5.9%), cannabis use (2.9%), chiropractic care for neck-related conditions (2.9%), and physiotherapy for elbow tendinopathy (2.9%). No intervention-related adverse events were reported in either group. Baseline characteristics were comparable between groups, with the exception of patient-reported disability (measured by the ODI) at baseline. Baseline disability scores were significantly greater in the aquatic therapy group (*p* = 0.008).

**Table 1:** Participant baseline characteristics

	Aquatic Therapy (*n* = 18)	Standard Care (*n* = 16)	*P*-Value
Age (y)	36.4 ± 10.4	37.4 ± 9.6	0.789
Female, *n* (%)	9 (50%)	10 (63%)	0.464
Height (cm)	171.4 ± 7.4	171.6 ± 11.0	0.971
Weight (kg)	77.8 ± 13.1	72.6 ± 14.0	0.275
BMI (kg/m^2^)	26.4 ± 3.8	24.6 ± 3.6	0.164
LBP NPRS (0–10)	5.1 ± 1.7	5.9 ± 1.5	0.188
ODI	31.7 ± 10.4	24.0 ± 3.5	0.008*
SF-12 Physical	35.6 ± 6.3	39.5 ± 4.7	0.050
SF-12 Mental	41.0 ± 10.6	46.1 ± 13.7	0.231
PCS	20.7 ± 10.0	16.8 ± 10.9	0.293
TSK	25.4 ± 5.2	22.6 ± 5.2	0.116
HADS	14.6 ± 6.0	11.6 ± 6.8	0.175
ISI	12.8 ± 3.7	11.1 ± 6.3	0.332

Values are presented as means ± standard deviations unless otherwise denoted

*BMI* Body mass index, *LBP* Lower back pain, *NPRS* Numerical Pain Rating Scale, *ODI* Oswestry Disability Index, *SF-12* 12-item Short Form Health Survey, *PCS* Pain Catastrophizing Scale, *TSK* Tampa Scale of Kinesiophobia, *HADS* Hospital Anxiety and Depression Scale, *ISI* Insomnia Severity Index

^*^p < 0.05

### Effects of aquatic therapy and standard care on muscle volume

The mixed model ANCOVA with repeated measures revealed no significant time*group interactions for the gluteus maximus, gluteus medius or gluteus minimus volume (all *p* > 0.05) while controlling for baseline disability. No significant change in the volume of the gluteus maximus, gluteus medius or gluteus minimus muscles was observed throughout the intervention for either group (Table 2).

**Table 2:** Adjusted (ODI) gluteal muscle volume means in the AT and SC groups

Variables	Measurement period	Aquatic Therapy *n* = 18	Standard Care *n* = 16	Main Effect of Group	Interaction effect between time and group
Gluteus Maximus Volume (cm^3^)	Baseline (Std. Error)	786.27 (46.18)	764.45 (49.29)	p-value = 0.91 F = 0.01 df = 1	*p*-value = 0.18 F = 1.92 df = 1
	10-weeks (Std. Error)	761.96 (46.38)	767.25 (49.51)		
	MD (95% CI)	-24.30 (-73.11 to 24.51)	2.80 (-49.30 to 54.90)		
	Main effect of time	*p*-value = 0.32 F = 1.03 df = 1	*p*-value = 0.91 F = 0.01 df = 1		
Gluteus Medius Volume (cm^3^)	Baseline (Std. Error)	321.93 (19.55)	333.11 (20.87)	*p*-value = 0.73 F = 0.12 df = 1	*p*-value = 0.88 F = 0.03 df = 1
	10-weeks (Std. Error)	316.16 (19.81)	325.33 (21.15)		
	MD (95% CI)	-5.78 (-22.50 to 10.94)	-7.78 (-25.63 to 10.07)		
	Main effect of time	*p*-value = 0.49 F = 0.50 df = 1	*p*-value = 0.38 F = 0.79 df = 1		
Gluteus Minimus Volume (cm^3^)	Baseline (Std. Error)	102.95 (5.72)	105.47 (6.11)	*p*-value = 0.71 F = 0.14 df = 1	*p*-value = 0.71 F = 0.14 df = 1
	10-weeks (Std. Error)	100.65 (5.63)	104.51 (6.01)		
	MD (95% CI)	-1.59 (-2.82 to 5.94)	-1.80 (-6.45 to 2.85)		
	Main effect of time	*p*-value = 0.32 F = 1.02 df = 1	*p*-value = 0.69 F = 0.69 df = 1		

*AT* Aquatic therapy group, *SC* Standard care group, *CI* Confidence interval

^*^ The mean difference is significant at the 0.05 level

### Effect of aquatic therapy and standard care on intramuscular fat (%IMF)

The mixed model ANCOVA with repeated measures revealed no significant time*group interactions for the gluteus maximus, gluteus medius or gluteus minimus intramuscular fat (all *p* > 0.05) while controlling for baseline disability. Postintervention, participants in the aquatic therapy group had greater decreases in intramuscular fat (mean difference [95% CI]) in the gluteus maximus (-2.56 [-4.47 to 0.64] %) and gluteus medius (-1.00 [-1.97 to 0.04] %) than those in the standard care group (Table 3). One outlier from the standard care group and aquatic therapy group was excluded from the postintervention intramuscular fat analysis because of data irregularities; however, their baseline intramuscular fat measurement was retained for analysis.

**Table 3:** Adjusted (ODI) gluteal muscle intramuscular fat means in the AT and SC groups

Variables	Measurement period	Aquatic Therapy *n* = 18	Standard Care *n* = 16	Main Effect of Group	Interaction effect between time and group
Gluteus Maximus %IMF^a^	Baseline (Std. Error)	16.18 (1.43)	12.80 (1.53)	*p*-value = 0.32 F = 1.02 df = 1	*p*-value = 0.08 F = 3.97 df = 1
	10-weeks (Std. Error)	13.63 (1.23)	13.12 (1.31)		
	MD (95% CI)	-2.56 (-4.47 to -0.64)*	0.32 (-1.72 to 2.36)		
	Main effect of time	*p*-value = 0.01 F = 7.43 df = 1	*p*-value = 0.75 F = 0.10 df = 1		
Gluteus Medius %IMF ^b^	Baseline (Std. Error)	12.96 (0.77)	11.52 (0.80)	*p*-value = 0.31 F = 1.05 df = 1	*p*-value = 0.54 F = 0.39 df = 1
	10-weeks (Std. Error)	11.95 (0.83)	10.97 (0.86)		
	MD (95% CI)	-1.00 (-1.97 to -0.04)*	-0.55 (-1.55 to 0.45)		
	Main effect of time	*p*-value = 0.04 F = 4.49 df = 1	*p*-value = 0.27 F = 1.28 df = 1		
Gluteus Minimus %IMF ^b^	Baseline (Std. Error)	13.60 (1.05)	13.36 (1.08)	*p*-value = 0.81 F = 0.06 df = 1	*p*-value = 0.11 F = 2.65 df = 1
	10-weeks (Std. Error)	12.87 (1.19)	13.93 (1.23)		
	MD (95% CI)	-0.74 (-1.81 to 0.33)	0.57 (-0.54 to 1.68)		
	Main effect of time	*p*-value = 0.17 F = 1.98 df = 1	*p*-value = 0.30 F = 1.10 df = 1		

*AT* Aquatic therapy group, *SC* Standard care group, *CI* Confidence interval

^a^ 1 missing data point from Standard Care group

^b^ 1 missing data point from Aquatic Therapy group

^*^ The mean difference is significant at the 0.05 level

### Effects of aquatic therapy and standard care on gluteal strength

The mixed model ANCOVA with repeated measures revealed no significant time*group interactions for gluteus maximus, gluteus medius or gluteus minimus strength (all *p* > 0.05) while controlling for baseline disability. Postintervention, both groups presented a significant increase in gluteal strength (mean difference [95% CI]): the aquatic therapy group presented improvements in the gluteus maximus (55.20 [31.84 to 78.55] Nm) and gluteus medius (72.34 [44.62 to 100.05] Nm), whereas the standard care group presented improvements in the gluteus maximus (41.45 [16.52 to 66.38] Nm) and gluteus medius (43.91 [14.32 to 73.49] Nm) (Table 4).

**Table 4:** Adjusted (ODI) gluteal muscle strength means in the AT and SC groups

Variables	Measurement period	Aquatic Therapy *n* = 18	Standard Care *n* = 16	Main Effect of Group	Interaction effect between time and group
Gluteus Maximus Mean Strength (Nm)	Baseline (Std. Error)	167.40 (14.20)	158.73 (15.16)	*p*-value = 0.50 F = 0.47 df = 1	*p*-value = 0.44 F = 0.61 df = 1
	10-weeks (Std. Error)	222.59 (17.12)	200.18 (18.28)		
	MD (95% CI)	55.20 (31.84 to 78.55)*	41.45 (16.52 to 66.38)*		
	Main effect of time	*p-*value = < 0.001 F = 23.24 df = 1	*p*-value = 0.002 F = 11.50 df = 1		
Gluteus Medius Mean Strength (Nm)	Baseline (Std. Error)	185.99 (15.49)	193.03 (16.54)	*p*-value = 0.79 F = 0.07 df = 1	*p*-value = 0.18 F = 1.85 df = 1
	10-weeks (Std. Error)	258.33 (21.42)	236. 94 (22.86)		
	MD (95% CI)	72.34 (44.62 to 100.05)*	43.91 (14.32 to 73.49)*		
	Main effect of time	*p*-value = < 0.001 F = 28.34 df = 1	*p*-value = 0.005 F = 9.16 df = 1		

*AT* Aquatic therapy group, *SC* Standard care group, *NM* Newton meters, *CI* Confidence interval

^*^ The mean difference is significant at the 0.05 level

### Correlation between changes in muscle morphology and patient-reported outcomes

The effects of each intervention on patient-reported outcomes have been previously reported in a separate manuscript [24], and are not described in detail in the present manuscript. Significant moderate correlations were revealed between gluteus maximus intramuscular fat and SF-12 physical health scores (*r* = − 0.33, *p* = 0.04) as well as between gluteus medius intramuscular fat and SF-12 physical health scores (*r* = − 0.31, *p* = 0.04). The correlations between changes in muscle morphology and patient-reported outcomes from baseline to 10 weeks are presented in Table 5.

**Table 5:** Correlations between changes in muscle morphology and clinical outcomes

Variables	ΔNPRS [95% CI]	ΔODI [95% CI]	ΔSF12-*P* [95% CI]	ΔSF12-M [95% CI]
ΔGMax Vol	0.07 [-0.24 to 0.39]	-0.05 [-0.37 to 0.26]	-0.15 [-0.47 to 0.16]	0.22 [-0.10 to 0.51]
ΔGMed Vol	0.09 [-0.24 to 0.40]	0.01 [-0.32 to 0.32]	0.05 [-0.27 to 0.36]	-0.01 [-0.33 to 0.31]
ΔGMin Vol	0.07 [-0.26 to 0.37]	-0.06 [-0.37 to 0.26]	-0.09 [-0.40 to 0.24]	0.12 [-0.22 to 0.41]
ΔGMax IMF	-0.01 [-0.33 to 0.31]	-0.05 [-0.36 to 0.29]	-0.33* [-0.62 to -0.04]	-0.02 [-0.35 to 0.29]
ΔGMed IMF ^a^	0.23 [-0.10 to 0.52]	0.11 [-0.21 to 0.42]	-0.31* [-0.60 to -0.007]	-0.21 [-0.52 to 0.09]
ΔGMin IMF ^a^	0.12 [-0.2 to 0.44]	0.14 [-0.20 to 0.44]	-0.19 [-0.50 to 0.12]	-0.23 [-0.54 to 0.07]

*ΔGMax Vol* changes in gluteus maximus volume; *ΔGMed Vol* changes in gluteus medius volume, *ΔGMin Vol* changes in gluteus minimus volume; *ΔGMax IMF* changes in gluteus maximus intramuscular fat, *ΔGMed IMF* changes in gluteus medius intramuscular fat; *ΔGMin IMF* changes in gluteus minimus intramuscular fat, *NPRS* Numerical Pain Rating Scale, *ODI* Oswestry Disability Index, *SF12-P* 12-item Short Form Health Survey – physical component, *SF12-M* 12-item Short Form Health Survey – mental component, *CI* Confidence interval

* Indicates *p* < 0.05

^a^ 1 missing data point

Significant moderate correlations were present between gluteus minimus intramuscular fat and pain catastrophizing scores (*r* = 0.39, *p* = 0.01). The correlations between changes in muscle morphology and psychosocial factors from baseline to 10 weeks are presented in Table 6.

**Table 6:** Correlations between changes in muscle morphology and psychosocial outcomes

Variables	ΔPCS ^a^ [95% CI]	ΔTSK [95% CI]	ΔHADS [95% CI]	ΔISI [95% CI]
ΔGMax Vol	-0.009 [-0.33 to 0.31]	-0.005 [-0.33 to 0.31]	-0.04 [-0.35 to 0.29]	-0.11 [-0.43 to 0.21]
ΔGMed Vol	-0.06 [-0.40 to 0.25]	-0.05 [-0.38 to 0.26]	-0.05 [-0.36 to 0.27]	-0.15 [-0.45 to 0.17]
ΔGMin Vol	-0.03 [-0.35 to 0.29]	-0.02 [-0.34 to 0.31]	-0.06 [-0.39 to 0.25]	-0.13 [-0.44 to 0.19]
ΔGMax IMF	0.07 [-0.25 to 0.38]	0.15 [-0.17 to 0.45]	0.20 [-0.10 to 0.51]	0.02 [-0.30 to 0.34]
ΔGMed IMF	0.25 [-0.08 to 0.53]	0.11 [-0.23 to 0.42]	0.20 [-0.10 to 0.52]	0.17 [-0.14 to 0.48]
ΔGMin IMF	0.39* [0.11 to 0.66]	0.13 [-0.19 to 0.45]	0.04 [-0.28 to 0.38]	0.23 [-0.09 to 0.53]

*ΔGMax Vol* changes in gluteus maximus volume, *ΔGMed Vol* changes in gluteus medius volume, *ΔGMin Vol* changes in gluteus minimus volume; *ΔGMax IMF* changes in gluteus maximus intramuscular fat, *ΔGMed IMF* changes in gluteus medius intramuscular fat, *ΔGMin IMF* changes in gluteus minimus intramuscular fat, *PCS* Pain Catastrophizing Scale, *TSK *Tampa Scale of Kinesiophobia, *HADS* Hospital Anxiety and Depression Scale, *ISI *Insomnia Severity Index, *CI* Confidence interval

* Indicates *p* < 0.05

^a^ 1 missing data point

### Correlations between changes in muscle morphology and strength

There were no significant correlations between gluteus muscle morphology and mean strength. The correlations between changes in gluteal muscle morphology and strength from baseline to 10 weeks are presented in Table 7.

**Table 7:** Correlations between changes in muscle morphology and changes in mean strength

Variables	ΔMean GMax Strength [95% CI]	ΔMean GMed Strength [95% CI]
ΔGMax Vol	-0.08 [-0.39 to 0.25]	-0.05 [-0.36 to 0.27]
ΔGMed Vol	-0.10 [-0.41 to 0.23]	-0.09 [-0.40 to 0.23]
ΔGMin Vol	-0.14 [-0.46 to 0.16]	-0.08 [-0.39 to 0.24]
ΔGMax IMF	0.04 [-0.29 to 0.35]	0.21 [-0.11 to 0.51]
ΔGMed IMF	0.02 [-0.30 to 0.35]	0.25 [-0.06 to 0.55]
ΔGMin IMF	0.03 [-0.29 to 0.36]	0.12 [-0.22 to 0.43]

*ΔGMax Vol* changes in gluteus maximus volume, *ΔGMed Vol* changes in gluteus medius volume, *ΔGMin Vol* changes in gluteus minimus volume, *ΔGMax IMF* changes in gluteus maximus intramuscular fat, *ΔGMed IMF* changes in gluteus medius intramuscular fat, *ΔGMin IMF* changes in gluteus minimus intramuscular fat, *Δmean GMax* *strength* changes in gluteus maximus mean strength, *Δmean GMed strength* changes in gluteus medius mean strength, *CI* Confidence interval

^*^ Indicates *p* < 0.05

### Effects of aquatic therapy and standard care on gluteal spatial distribution

**Fig. 2:**
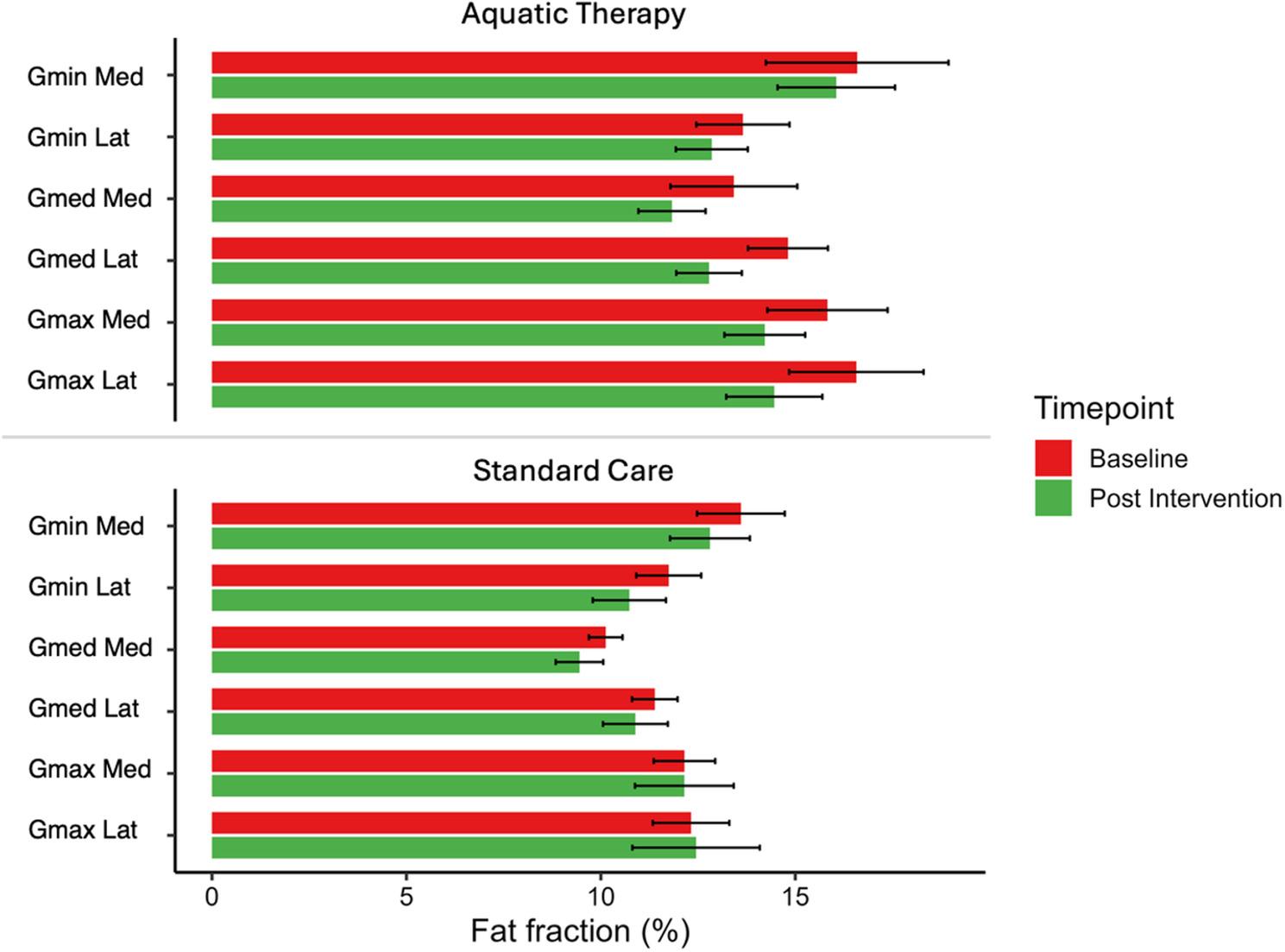
Gluteal muscle IMF spatial distribution (medial/lateral) means

No significant changes were observed in the medial or lateral spatial distributions of the gluteus minimus, gluteus medius or gluteus maximus muscles in either group from baseline to postintervention (Figs. 2 and 4).

* *p* > 0.05; AT, aquatic therapy; SC, standard care; Gmin Med, Gluteus minimus medial spatial distribution; Gmin Lat, Gluteus minimus lateral spatial distribution; Gmed Med, Gluteus medius medial spatial distribution; Gmed Lat, Gluteus medius lateral spatial distribution; Gmax Med, Gluteus maximus medial spatial distribution; Gmax Lat, Gluteus maximus lateral spatial distribution.

**Fig. 3:**
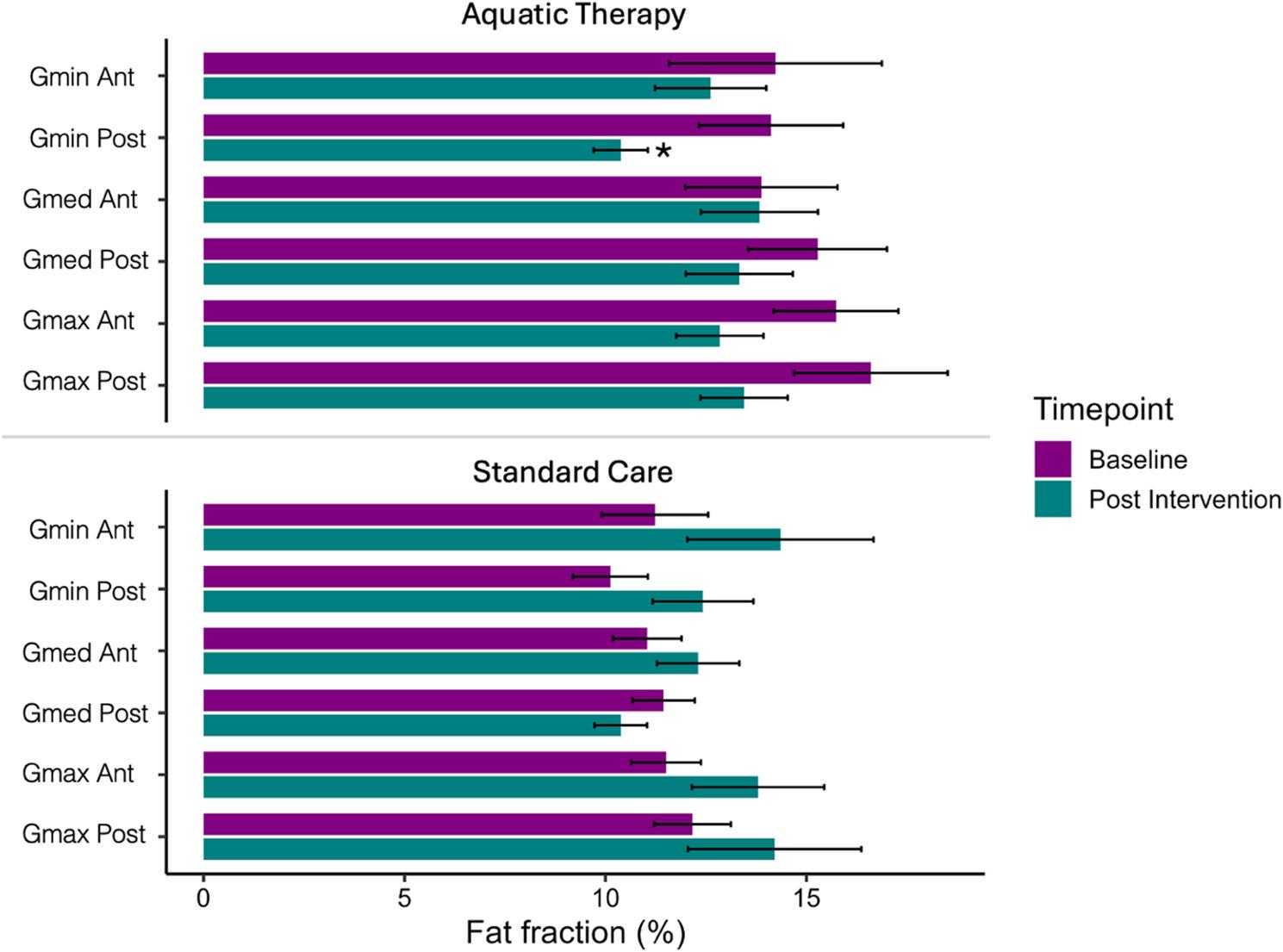
Gluteal muscle IMF spatial distribution (anterior/posterior) means

**Fig. 4:**
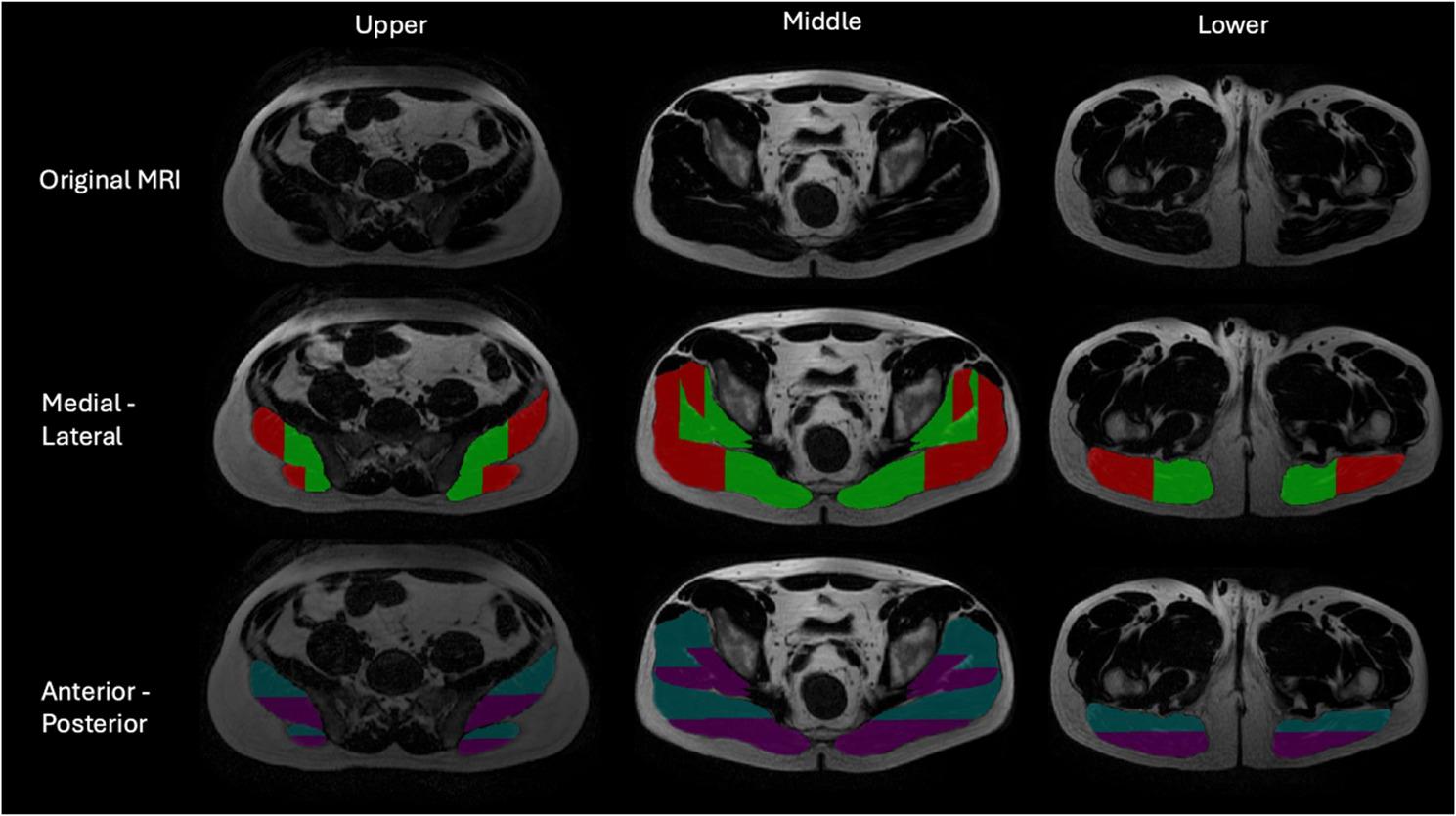
Gluteal musculature spatial distribution tilings (2D)

No significant changes in anterior or posterior spatial distribution were observed from baseline to postintervention in the gluteus medius or gluteus maximus muscles in either group. A significant decrease in intramuscular fat was observed only in the posterior portion of the gluteus minimus from baseline to postintervention in the aquatic therapy group (*p* = 0.04). A single comparison between two tiles (i.e., anterior/posterior) was performed for each gluteal muscle independently; no correction for multiple comparisons was necessary (Figs. 3 and 4).

* *p* > 0.05; AT, aquatic therapy; SC, standard care; Gmin Ant, Gluteus minimus anterior spatial distribution; Gmin Post, Gluteus minimus posterior spatial distribution; Gmed Ant, Gluteus medius anterior spatial distribution; Gmed Post, Gluteus medius posterior spatial distribution; Gmax Ant, Gluteus maximus anterior spatial distribution; Gmax Post, Gluteus maximus posterior spatial distribution.

2D: two-dimensional; medial (green); lateral (red); anterior (blue); posterior (purple)

## Discussion

### Effects of aquatic therapy and standard care on muscle volume

Our findings revealed no significant time-by-group interaction effects for gluteus maximus, medius, or minimus muscle volume. Additionally, there were no significant within-group changes in muscle volume over the 10-week intervention period for either the aquatic therapy or standard care groups. While exercise interventions are frequently associated with neuromuscular adaptations [49], muscle morphological changes may require longer durations and higher intensity loading, with an initial focus on adequate muscle recruitment [50]. Emerging evidence from a recent RCT suggested that targeted, higher-intensity, and longer-duration interventions are more likely to elicit significant changes in paraspinal muscle composition, with adaptations occurring primarily at upper lumbar levels rather than uniformly across the spine [23]. Similarly, the gluteal muscles may require greater intensity and longer duration for morphological changes to become evident.

While the changes in volume were not statistically significant, there was a trend toward a decrease in overall gluteal muscle volume, accompanied by a concomitant decrease in intramuscular fat. Similar trends have been reported in lower paraspinal muscles, suggesting that longer-duration interventions may be necessary to produce significant changes in muscle composition [23]. Previous studies have noted that muscle hypertrophy is more likely to occur after 12 or more weeks of progressive resistance training [51], which may explain the absence of significant changes in muscle volume observed in the present study.

Another possible explanation is that aquatic therapy intervention, while beneficial for reducing joint loading and allowing for movements that would otherwise be challenging to perform on land [2, 52], may not have provided sufficient mechanical overload to stimulate hypertrophic changes [53]. Buoyancy reduces the effect of gravity and, consequently, potentially muscle activation in muscles such as the gluteal muscles [2]. Although buoyancy and hydrostatic pressure may facilitate early-stage rehabilitation, they may limit the stimulus required for morphological adaptation in muscle tissue [52].

Additionally, many studies have assessed hypertrophy via cross-sectional area from a single slice [54–57], whereas 3D muscle volume, as used in the present study, provides a more comprehensive assessment of overall muscle morphology. Further research investigating muscle volume changes across interventions is needed to understand how muscle morphology adapts in response to exercise.

### Effect of aquatic therapy and standard care on intramuscular fat (%IMF)

Although no significant group-by-time interactions were observed, participants in the aquatic therapy group demonstrated significantly greater reductions in the intramuscular fat of the gluteus maximus and gluteus medius muscles over the 10-week intervention period. Specifically, the aquatic therapy group presented a mean decrease of 2.56% (95% CI: -4.47 to -0.64) in the gluteus maximus intramuscular fat content and 1.00% (95% CI: -1.97 to -0.04) in the gluteus medius intramuscular fat content, whereas the changes in the standard care group were not statistically significant.

To contextualize the magnitude of the observed changes in IMF, the standard error of measurement (SEM) and minimal detectable change (MDC) were estimated using previously reported reliability coefficients for MuscleMap-based segmentation of the gluteal muscles [35]. The calculated MDC values were 1.89% for the gluteus maximus, 3.19% for the gluteus medius, and 1.89% for the gluteus minimus. In the aquatic therapy group, the reduction in gluteus maximus IMF (2.56%) exceeded the estimated MDC, suggesting that the change likely reflects a physiological adaptation beyond measurement error. In contrast, all other observed changes in gluteal muscle outcomes in both the aquatic therapy and standard care groups were below their respective MDC thresholds, indicating that they fall within expected measurement error and should therefore be interpreted with caution.

From a biomechanical standpoint, the gluteus maximus and medius primarily contribute to hip extension, hip abduction, external rotation, and internal rotation. These movement patterns were specifically addressed throughout the entire 10-week aquatic therapy intervention. In contrast, the standard care group received treatment based on the initial assessment, potentially resulting in a lower volume and insufficient exposure to observe a change in gluteal intramuscular fat. The absence of significant changes in the gluteus minimus %IMF across both groups could be due to its deeper anatomical position and lower recruitment in common exercise movements, including those performed in aquatic environments [58].

Evidence on whether exercise can reverse muscle intramuscular fat in individuals with chronic low back pain is limited. A recent systematic review revealed that exercise may not reverse paraspinal muscle intramuscular fat in individuals with chronic low back pain [59]. However, emerging evidence from a recent RCT suggested that adaptations in paraspinal muscle composition may vary by spinal level, indicating that targeted interventions could produce site-specific rather than uniform changes [23]. Consequently, larger, higher-quality studies are needed to clarify the effects of exercise on lumbopelvic muscles.

### Effects of aquatic therapy and standard care on gluteal strength

The gluteal muscles play a key role in force transfer, pelvic stability, and lumbar support, making them a common target in chronic low back pain rehabilitation through strengthening and motor control exercises [60]. Both the aquatic therapy and standard care groups demonstrated significant within-group improvements in gluteus maximus and gluteus medius muscle strength over the 10-week intervention period. However, no significant time x group interaction was observed. The participants in the aquatic therapy group showed mean improvements of 55.20 Nm (95% CI: 31.84 to 78.55) in gluteus maximus strength and 72.34 Nm (95% CI: 44.62 to 100.05) in gluteus medius strength. Similarly, the standard care group experienced strength improvements of 41.45 Nm (95% CI: 16.52 to 66.38) and 43.91 Nm (95% CI: 14.32 to 73.49) in the gluteus maximus and medius, respectively.

According to previous studies investigating gluteal muscle strength before and after interventions for chronic low back pain, improvements in the gluteus maximus ranged between 9.31 and 26.17 N (10.36-30% increase), whereas gains in the gluteus medius ranged between 6.52 and 24.40 N (9-32.05% increase) [61–63]. However, it is important to note that these studies were limited by relatively short intervention durations of only 4–6 weeks. In our study, both groups improved, but the aquatic therapy group demonstrated greater gains in gluteal muscle strength, likely due to consistent resistance-based exercises targeting the gluteal muscles throughout the 10-week intervention. By comparison, the standard care intervention was individualized based on assessment findings and may not have included targeted gluteal training across the full duration.

Psycharakis et al. (2019) reported that during unilateral lower limb exercises such as hip abduction, extension, and single-leg squats, gluteal muscle activity was equal to or greater on land than on water [2]. Buoyancy and hydrostatic pressure in water reduce muscular demands by assisting movement and providing postural support, resulting in lower gluteal activation than that in land, where muscles need to overcome gravitational forces [22]. Given the significantly greater baseline disability in the aquatic therapy group, the supportive properties of water likely allowed participants to perform movements more consistently and effectively from an earlier stage, improving gluteal activation throughout the intervention.

Interestingly, the strength improvements occurred in the absence of significant muscle volume changes, suggesting that neural adaptations, such as increased motor unit recruitment and muscle coordination, may have been the primary drivers of improvement [49].

### Correlation between changes in muscle morphology and changes in patient-reported outcomes and strength

Notably, there was a significant moderate negative correlation between reductions in intramuscular fat of the gluteus maximus and medius muscles and improvements in physical health-related quality of life, as measured by the SF-12 physical component score (*r* = -0.33 and − 0.31, respectively; both *p* = 0.04). Given the role of the gluteal muscles in pelvic stability, force transfer, and lumbar support [60], reductions in intramuscular fat may improve movement efficiency and reduce mechanical stress on the spine, thereby contributing to improved physical function and well-being.

In addition, a significant moderate positive correlation was identified between changes in gluteus minimus intramuscular fat and pain catastrophizing scores (*r* = 0.39, *p* = 0.01), indicating that increased intramuscular fat in this muscle may be associated with increased levels of pain-related cognitive distress. The gluteus minimus, although often overlooked compared with the larger gluteal muscles, plays a key role in hip stabilization and load transfer during gait and postural control [64]. Increased intramuscular fat in this muscle may reflect greater disuse or neuromuscular inhibition, which could contribute to altered biomechanics and reinforce pain-related fear or avoidance behaviours [65, 66]. Conversely, persistent pain catastrophizing may also limit engagement in movement or rehabilitation, thereby impeding muscle quality improvements [67]. There is value in assessing muscle quality (i.e., intramuscular fat) as a sensitive marker of functional and psychosocial improvement in chronic low back pain populations. Further longitudinal studies are needed to determine whether directly targeting muscle composition can accelerate improvements in patient-reported outcomes.

Despite these associations, no significant correlations were found between changes in gluteal muscle morphology (i.e., volume or composition) and changes in muscle strength. Early strength improvements are often driven by neural adaptations rather than morphological changes [49]. As such, improvements in muscle composition may reflect broader recovery processes not directly captured by strength metrics alone. However, prior research revealed a positive relationship between gluteal muscle volume and strength in individuals with hip/groin pain, whereas no significant relationship was found between intramuscular fat volume and strength [68]. Therefore, further research is needed to fully understand the relationship between strength and muscle morphology in chronic pain populations.

Exploratory analyses were conducted to assess changes in patient-reported outcomes from baseline to postintervention. The participants in both groups demonstrated significant improvements in patient-reported outcomes; however, these results were published in a separate manuscript [24].

### Effects of aquatic therapy and standard care on gluteal spatial distribution

A significant decrease in intramuscular fat was observed in the posterior portion of the gluteus minimus in the aquatic therapy group from baseline to post-intervention (*p* = 0.04), suggesting a localized and muscle-specific improvement in muscle quality. The posterior portion of the gluteus minimus may have been particularly responsive to aquatic therapy due to the unloading effects of buoyancy, which reduce gravitational stress on the body and allow for greater ease of movement and muscle engagement without exacerbating pain [22, 45]. Kivle et al. (2018) reported that patients with end-stage hip osteoarthritis exhibited sparse to moderate fatty degeneration of the gluteus minimus [69]. A similar concept may apply to individuals with chronic low back pain, who often exhibit elevated levels of intramuscular fat. A greater baseline level of muscle degeneration may provide greater capacity for improvement through targeted rehabilitation interventions.

In contrast, no significant regional differences in intramuscular fat were observed across the anterior-posterior or medial-lateral regions of the gluteus medius and maximus. This may suggest that these muscles may not follow a compartmentalized pattern of fat change but rather exhibit more uniform or diffuse intramuscular adaptations. The diffuse patterns may reflect the complex architecture and multiplanar functions of the gluteus medius and maximus. A study investigating changes in intramuscular fat demonstrated that prolonged unloading (bed rest) led to region-specific (i.e., upper, lower) increases in fat within the gluteus maximus [36]. However, no such spatial patterns were observed in the gluteus medius or minimus muscles. Therefore, further research is needed using advanced imaging and muscle segmentation techniques to better understand regional versus global adaptations in muscle composition in response to rehabilitation strategies.

### Limitations

Several limitations should be considered when interpreting the results of this study. First, as this study examined a secondary objective of a parent RCT, it was not prospectively powered for the specific outcomes of this manuscript. As a result, the sample size was modest and may have limited the ability to detect small between‑group differences. Second, owing to resource constraints, the same assessors who conducted postintervention testing also administered the interventions, resulting in a lack of blinding. This could have introduced potential bias in the data collection and assessment. Future studies should implement assessor blinding to minimize this risk and enhance the reliability of outcome measures. Third, although the intervention duration of 10 weeks aligns with prior research on chronic low back pain, this timeframe may not have been sufficient to elicit detectable changes in muscle volume, especially in populations with long-standing muscular dysfunction. Fourth, participants in the aquatic therapy group did not receive a physical examination, which may have affected the therapeutic alliance between participants and therapists. Standardizing therapist interactions across interventions in future studies may help control for these potential effects. Additionally, the use of surface-based strength testing does not fully isolate the gluteal muscle and may have been influenced by synergist activation or movement compensation. Finally, although the anterior-posterior and medial-lateral tile-based approaches for assessing spatial distribution have previously demonstrated reliability [70], they may not fully account for the oblique orientation, morphology and action of the gluteal muscles. With ongoing technological progress, future research is expected to adopt more advanced image analysis methods, such as template-based three-dimensional voxelwise statistical parametric mapping [71]. Template-based approaches have already shown value by enabling accurate, reproducible, and detailed spatial assessments of the spinal cord [72] and paraspinal muscles [73]. In addition, principal component analysis-based orientation frameworks, as recently applied for determining intervertebral disc planes and height [74], could further facilitate anatomically consistent partitioning of paraspinal muscles into medial-lateral and superficial-deep compartments.

### Future directions

Future research should consider longer intervention durations with progressive overload strategies to better capture potential hypertrophic adaptations in the gluteal muscles. Studies with larger, more diverse samples are needed to enhance generalizability and to allow for subgroup analyses on pain duration, age, sex, or psychological profile. Incorporating longitudinal follow-up data will also be essential for determining whether short-term neuromuscular improvements translate into long-term functional recovery and reduced recurrence. Future work should explore the mechanistic relationship between muscle composition and psychosocial outcomes, potentially informing more personalized and biopsychosocial integrated treatment plans. Finally, future research should refine spatial mapping methods to capture the oblique orientation and complex architecture of the gluteal muscles.

## Conclusion

Both aquatic therapy and standard care interventions led to significant improvements in gluteal muscle strength among individuals with chronic low back pain, although no significant differences were found between the groups. While no changes in gluteal muscle volume were observed, the aquatic therapy group presented significant reductions in intramuscular fat of the gluteus maximus and medius, findings that were moderately correlated with improved physical quality of life. These results suggest that aquatic therapy may confer unique benefits to muscle composition and psychosocial well-being, even when morphological changes in muscle size are not present. Overall, these findings reinforce the value of exercise-based interventions for chronic low back pain and highlight the importance of targeting both physical and psychological domains in rehabilitation strategies.

## Supplementary Information

Supplementary Material 1.

## Data Availability

All extracted data for the current study are available from the corresponding author upon reasonable request.

## Abbreviations

cLBP: chronic low back pain
ANCOVA: Analysis of covariance
RCT: Randomized controlled trial
NPRS: Numerical Pain Rating Scale
ODI: Oswestry Disability Index
SF-12: Short-Form 12
PCS: Pain Catastrophizing Scale
TSK: Tampa Scale of Kinesiophobia
HADS: Hospital Anxiety and Depression Scale
ISI: Insomnia Severity Index
MRI: magnetic resonance imaging
IMF: Intramuscular fat
3D: Three-dimensional
CI: Confidence interval
