# Supplementary material for: Effects of aquatic therapy vs. standard care on gluteal muscle morphology and function in individuals with chronic low back pain: a randomized controlled trial
Source: BMC Sports Sci Med Rehabil. 2026 Apr 6;18:241. doi: 10.1186/s13102-026-01666-0 (PMC13192150; doi:10.1186/s13102-026-01666-0)
Supplement: Supplementary file 1 — Supplementary Material 1. [file 13102_2026_1666_MOESM1_ESM.docx]

**Supplementary Table 1.** Aquatic therapy program

|  | **Aquatic Exercises** | **Volume** |
| --- | --- | --- |
| **Warm-Up**  (10 minutes) | **Water walking:** forwards, backwards, sideways - left and right | 2 minutes (30s / direction) |
|  | **Small hops:** feet lateral - back to neutral + alternating front to back hops | 2 min (1 min / direction) |
|  | **Leg kicks:** kick front, lateral, back | 1 min (30s / leg) |
|  | **Stationary jogging:** raise knees, heel to buttocks - as tolerated | 2 minute (1 min each) |
|  | **Horizontal kicking:** Prone and supine (holding onto edge of pool) | 1 minute (30s each) |
|  | **Upper body oscillations:** Moving both arms in oscillatory fashion from horizontal adduction to horizontal abduction | 1 minute |
|  | **Bow and Arrow:** shoulder horizontal abduction and contralateral row, alternating sides | 1 minute |
| **Training**  **Session**  (​​40 minutes) | **Multi-directional Lunges:** (forward, backward, sideways) on step in water | 2 sets x 10 repetitions / direction |
|  | **Squats:** Place both arms by sides with forearms pronated. Flex knees to squat while bringing arms together just below the water surface, and then return to starting position. Use underwater steps to progress exercise. | 2 sets x 10 repetitions |
|  | **Step-Ups:** Using underwater step, place one foot on the step with hands on your hips and transfer weight to step up. | 2 sets x 10 repetitions |
|  | **Single leg squat:** Stand on one leg with both arms crossed at chest while placing one knee in 90-degree flexion. Perform single leg squat on other limb until the knee moves in front of the toes. Modification: hold onto pool railing | 2 sets x 10 repetitions each side |
|  | **Standing Hip Abduction:** Stand on one leg while arms slightly abducted and abducting the other leg as far as possible while attempting to keep a neutral position throughout the motion. Use elastic band above ankles to increase resistance. | 2 sets x 10 repetitions each side |
|  | **Standing Hip Extension:** Stand on one leg while arms slightly abducted and extending the other hip as far as possible while attempting to keep a neutral position throughout the motion. Use elastic band above ankles to increase resistance. | 2 sets x 10 repetitions each side |
|  | **Concentric chest press + row:** Stand up in chest-deep water and hold kickboard vertically between hands with arms fully outstretched in front just below water surface. Pull kickboard close to chest (bringing arms backwards towards body) and then push forwards to starting position, while staying put. | 2 sets x 10 repetitions |
|  | **Underwater punches:** Have arms just below water surface, with one arm fully outstretched in front and the other close to body. Holding Aqualogix dumbbells, perform alternate punching with each arm. | 2 sets x 10 repetitions |
|  | **Concentric Shoulder Flexion + Shoulder Extension:** Place one arm by side and other outstretched in front just below water surface with palms facing direction of movement. Alternately bring one arm upwards just below water surface while bringing the other arm to the side. Use hand paddles to increase resistance. | 2 sets x 10 repetitions |
|  | **Knee Raises:** Hold dumbbell floats in each hand with arms by sides. Raise both knees alternately until thighs are parallel to the surface of the water. | 2 sets x 10 repetitions |
|  | **Woodchopper:** Have hands with arms fully outstretched in front just below water surface. Rotate trunk gradually as far to one side as possible and then back to the midline. Alternately do the same with other side. | 2 sets x 10 repetitions each side |
|  | **Water Ab Rollout:** With a band wrapped around the trunk, start in upright posture with arms outstretched and hands resting on the surface holding dumbbell floats. Therapist hold band and the patient slowly moves dumbbell floats forwards while keeping the body in a neutral posture tilting on the tips of the toes, and then return to starting position while maintaining a neutral spine. | 2 sets x 10 repetitions |
| **Cool-Down**  (10 minutes) | **Free water-activity:** walking, standing, swimming | 1 minute |
|  | **Stretching/Mobility exercises:**   - Hip CARs (controlled articular rotations) - Knee-to-wall (ankle mobility) - Knee-to- chest hold - Standing quad stretch - Standing hamstring stretch (extend leg + lean in) - Anterior chain stretch: grab arms behind back + open up chest - Standing figure 4 stretch - Standing open book (hold onto side of pool, lift leg + rotate away) | 3 x 20s per muscle group |
|  | **Relaxation:** deep diaphragmatic breathing - supine with noodle supporting them | 5-6 breaths (1 minute) |
